# 患者报告结局和医患共同决策在胸外科临床中的应用

**DOI:** 10.3779/j.issn.1009-3419.2024.106.26

**Published:** 2024-10-20

**Authors:** Weihao CHEN, Mengni ZHANG, Cheng SHEN

**Affiliations:** ^1^610041 成都，四川大学华西医院临床医学院; ^1^West China School of Medicine, West China Hospital, Sichuan University, Chengdu 610041, China; ^2^610041 成都，四川大学华西医院临床医学院，病理科; ^2^Department of Pathology, West China Hospital, Sichuan University, Chengdu 610041, China; ^3^610041 成都，四川大学华西医院临床医学院，胸外科; ^3^Department of Thoracic Surgery, West China Hospital, Sichuan University, Chengdu 610041, China

**Keywords:** 胸外科, 共同决策, 患者报告结局, 医患关系, Thoracic surgery, Shared decision-making, Patient-reported outcomes, Doctor-patient relationship

## Abstract

胸外科涵盖了肺癌、食管癌、纵隔肿瘤等多种胸部疾病的诊断和治疗，这些疾病的治疗方案复杂，常涉及手术、化疗和放疗等多种方式，每种方式对患者生活质量有不同影响。患者报告结局（patient-reported outcomes, PRO）和医患共同决策（shared decision-making, SDM）在这一领域越来越重要。PRO使患者能够直接报告健康状况和治疗影响，帮助医生调整治疗计划；SDM则通过信息共享和共同决策，确保治疗方案符合患者的个人价值观和偏好。综合运用PRO和SDM可提高患者满意度和治疗效果，但也面临数据收集、时间管理等挑战。未来研究应集中于开发更高效的PRO工具和SDM流程，来提升以患者为中心的医疗服务质量。

## Abstract (en)

Thoracic surgery encompasses the diagnosis and treatment of various chest diseases such as lung cancer, esophageal cancer, and mediastinal tumors. The treatment plans for these diseases are complex and often involve a combination of surgery, chemotherapy, and radiotherapy, each with different impacts on the patient's quality of life. Patient-reported outcomes (PRO) and shared decision-making (SDM) are becoming increasingly important in this field. PRO allows patients to directly report their health status and the effects of treatment, aiding doctors in adjusting treatment plans. SDM ensures that treatment plans align with the patient's personal values and preferences through information sharing and joint decision-making. The comprehensive application of PRO and SDM can enhance patient satisfaction and treatment outcomes, though it also faces challenges such as data collection and time management. Future research should focus on developing more efficient PRO tools and SDM processes to improve patient-centered healthcare quality.

胸外科是一个涉及诊断和治疗胸部疾病的医疗领域，包括但不限于肺癌、食管癌、纵隔肿瘤、胸膜疾病和胸腺疾病等。这些疾病往往伴随着复杂的治疗方案，如手术、化疗、放疗或进行各种治疗方案的组合，每种治疗方式都有其独特的风险、益处和对患者生活质量的影响。患者在面对治疗选择时，不仅要考虑生存率和预后，还要考虑自身对于各种治疗方案的接受度。

在这一背景下，患者报告结局（patient-reported outcomes, PRO）^[1]^和医患共同决策（shared decision-making, SDM）^[2]^的概念逐渐兴起，并在胸外科发挥着日益重要的作用。PRO使患者能够直接报告其健康状况、症状、治疗副作用以及生活质量，这些信息对于理解疾病和治疗如何影响患者日常生活至关重要^[3]^。PRO的数据可以帮助医生监测治疗效果，调整治疗计划，并在临床试验中评估新疗法的效果。SDM则是一种理想的决策过程，它强调医生和患者之间的信息共享、讨论以及共同决定最佳治疗方案^[4]^。在胸外科SDM可以帮助患者在充分了解自己的健康状况和治疗选择的基础上，做出与其个人价值观和偏好相符合的决策^[5]^。

将PRO和SDM融入胸外科的临床实践，不仅能够提高患者的治疗满意度和生活质量^[6]^，还能够优化资源分配，提高医疗服务的整体效率^[7]^。因此，探讨PRO和SDM在胸外科中的应用，对于推动以患者为中心的医疗服务和提升治疗结果具有重要的理论和实践意义^[8]^。

## 1 PRO和SDM在临床中应用优缺点及关系

### 1.1 PRO和SDM的相互关系

2009年美国食品药品监督管理局（Food and Drug Administration, FDA）对PRO的定义为直接来自患者对自身健康状况、功能状态以及治疗感受的报告，不包括医护人员及其他任何人员的解释^[9]^。PRO可以包括：（1）症状的严重性：胸外科患者常面临多种症状，如疼痛、呼吸困难、咳嗽和胸闷等，PRO可以帮助患者详细报告这些症状的严重性，指导医生制定更有效的治疗方案；（2）治疗的副作用：胸外科治疗常伴随多种副作用，如恶心、呕吐、疲劳和感染等，PRO可以帮助患者详细报告这些副作用的严重性，从而帮助医生调整治疗方案；（3）日常活动的能力：胸外科患者常面临日常活动能力的下降，如行走、爬楼梯和家务活动等，通过采用PRO可以帮助患者详细了解这些活动的能力，助力康复计划的稳步推进；（4）心理状态以及生活质量等多个维度^[10]^。SDM是一种医疗决策过程，医生和患者共同参与，基于患者的个人价值观和偏好以及医生的专业知识来共同决定治疗方案^[5]^。SDM强调医生提供所有可行的治疗选项和它们的相关信息，患者则在理解这些信息的基础上，参与到最终的治疗选择中^[11]^。

PRO和SDM之间存在着密切的互动关系。PRO为SDM提供了基础数据，使医生能够了解患者的个人经历、治疗反应和生活质量的改变^[12]^，这些信息是SDM中不可或缺的部分。患者的这些直接报告有助于医生更全面地理解患者的需求和期望，从而在SDM过程中提供个性化的治疗建议。在SDM中PRO的作用不仅局限于提供患者自身的信息。它还能够增强患者的自主性，让患者在决策过程中扮演更加积极的角色。当患者看到自己的反馈被医疗团队认真对待时，他们可能会更积极地参与到治疗过程中，提高治疗依从性^[13]^。此外，PRO的使用也促进了医生与患者之间的沟通，为开展SDM奠定了一个更加坚实的信任基础。

PRO和SDM在以患者为中心的医疗模式中相辅相成，共同作用于提高医疗质量和患者满意度。PRO的有效利用不仅能够丰富SDM的内容，还能够促进医患之间的沟通，使双方在更真实的基础上共同决策，实现最优的治疗结果^[14]^。

### 1.2 PRO在胸外科的应用及其优点和不足

在胸外科领域，PRO的应用已经逐渐成为评估治疗效果和患者生活质量的一个重要组成部分。具体而言，PRO在胸外科中的应用可以体现在几个方面^[3]^。首先，它被用于监测患者术后的恢复情况，包括疼痛管理、肺功能的恢复以及日常活动能力的改善。其次，PRO用于评估长期治疗方案，如化疗或放疗对患者生活质量的影响。此外，PRO数据也被广泛用于临床试验中，以评估新治疗方法对患者的实际影响。

PRO的应用具有显著的优点：（1）PRO增强了患者的声音，使医生能够从患者的视角了解治疗效果，有助于医疗团队更好地理解患者的需求和偏好^[15]^；（2）PRO的使用有助于改善预后，通过持续监测患者的反馈，医生可以及时调整治疗方案，预防并管理潜在的并发症^[16]^；（3）PRO数据的收集和分析可以帮助医疗团队识别治疗过程中的问题，从而不断优化治疗流程和提高患者的整体满意度^[17]^。

尽管如此，PRO的应用也存在一些局限性。卢羿同等^[3]^通过检索Web of Science等核心数据库中相关文献发现，在现今对肺癌患者的PRO相关研究中所涉及的患者疗效的评估指标较少，对于PRO在肺癌患者中的应用有效性提供的理论依据有限，评判标准不一^[1]^。首先，数据解释的主观性是一个挑战，由于PRO是基于患者的个人感受和评价，不同患者对相同症状的感知可能存在差异，这可能影响数据的一致性和可比性。其次，实施PRO的难度也不容忽视，有效地收集、管理和分析PRO数据需要专业的工具和系统，而这可能会带来额外的成本和资源需求，对于这一问题，李春阳等^[18]^通过对文献数据库中国内外应用于慢性阻塞性肺疾病（chronic obstructive pulmonary disease, COPD）急性加重期PRO的23种测评工具进行分析提出，可以通过开发新的更合适的测评工具，联合应用经典与新时代的测量理论，通过统计学方法减少测量误差等方法为更好地处理PRO收集到数据提供抓手。此外，医生和患者都需要对PRO的重要性有充分的认识，以确保数据收集的质量和完整性。虽然PRO在胸外科的应用带来了多方面的优势，但在实际操作中仍需克服一些障碍，以实现其在临床实践中的最大潜力。

### 1.3 SDM在胸外科的应用及其优点和不足

SDM在胸外科的应用主要体现在患者面临多种治疗选项时的决策过程中。例如，在选择肺癌治疗方案时患者可能需要在手术、放疗、化疗或是这些方法的组合中做出选择^[19]^。SDM使得患者能够在充分了解各种治疗方法的潜在利益和风险后，根据自己的偏好和生活质量考虑后与医生共同决定最合适的治疗路径^[20]^。

SDM的应用存在多方面的优点：（1）SDM显著提高了患者的满意度，因为患者感到自己的意见在治疗过程中得到了听取和尊重，患者的个人情况和需求得到了保障；（2）SDM能够改善患者依从性，因为在疑惑得到医生的详细解释后，患者对于共同决定的治疗计划往往更乐意执行；（3）SDM还有助于减少不必要的医疗干预，在医患双方综合评价患者的疾病进展和身体情况后，能够制定出更倾向于选择符合其个人价值观和生活质量考虑的治疗方法，避免过度医疗^[21]^。

SDM在胸外科的应用也存在一些局限性：（1）SDM的过程往往比传统的医疗决策模式更加消耗时间，因为它需要医生花费额外的时间来解释治疗选项并与患者讨论，基于医疗资源分布时间和空间不均匀的现状，实现SDM的普适化是一个新的难题；（2）由于患者的医学素养参差不齐，不同患者的文化水平和自主性不同，加之外界信息对患者的选择做出干扰等，患者期望参与诊疗决策的意愿并不乐观^[22]^；（3）医生的个人偏好和患者的情感状态也可能影响决策过程，导致决策结果偏离患者最初的意愿。

总的来说，SDM在胸外科中的应用促进了医患之间的沟通和信息交流，有助于形成更加个性化和以患者为中心的治疗方案。然而，为了充分发挥SDM的优势，需要在实践中解决时间管理、教育培训以及信息平衡等方面的挑战。

## 2 PRO和SDM在胸外科临床中的应用

### 2.1 应用现状

胸外科疾病通常包括了一系列复杂且多样的病种，如肺癌、食管癌、纵隔肿瘤等，这些疾病的特点在于它们往往涉及到高风险的手术治疗，同时这些疾病也很容易迁延成为慢性疾病，对患者的生活质量有着显著影响，并且可能需要长期的治疗过程和跟踪。例如，肺癌手术后患者可能需要面对肺功能的减弱，食管癌患者可能会经历吞咽困难，这些都是在治疗时需要考虑的因素。在这样的背景下，PRO和SDM的应用显得尤为重要。PRO可以帮助医疗团队监测和评估患者的症状、副作用以及治疗对日常生活的影响，从而对治疗方案进行适时调整^[23]^。在肺癌治疗中PRO可以用来评估手术、放疗或化疗对患者生活质量的具体影响，以及患者对于不同治疗方案的偏好，而SDM在胸外科疾病治疗中的应用则体现在医生和患者共同讨论并决定最佳治疗路径上。考虑到胸外科疾病的高风险性和对生活质量的影响，SDM能够确保患者在充分理解可能的治疗结果和风险的基础上，参与到治疗决策中^[24]^。例如，在食管癌的治疗中患者可能需要在手术和辅助化疗之间做出选择，SDM可以帮助患者根据自己的生活质量目标和对副作用的容忍度做出更适合自己的决定。

结合具体案例来看，韩港飞等^[25]^通过对313例慢性心力衰竭的住院患者的健康期望影响因素进行一般线性回归分析，证明了通过使用PRO和健康期望评估，可以帮助医生筛查出患者在各个方面和层面需要重点干预的领域，从而指导并实现SDM，提高患者的治疗依从性，最终能够获得更好的治疗效果。通过PRO的应用，医生能够了解患者在日常生活中的功能限制和治疗偏好，在此基础上进行SDM可以携手制定出最合适的治疗方案，不仅考虑治疗效果，也考虑患者的生活质量和心理状态。

胸外科疾病的治疗需要综合考虑多种因素，PRO和SDM的应用能够帮助医疗团队和患者共同面对这些挑战，提高治疗的个性化程度和整体满意度。当然，这也要求相关医疗团队对PRO的收集和分析有足够的重视，并在SDM过程中投入足够的时间和精力，以确保做出最佳的治疗决策。

### 2.2 PRO和SDM的应用总述

PRO和SDM在多种疾病中均有涉及，例如慢性心力衰竭、哮喘、COPD等。在胸外科中，主要应用于肺癌、食管癌等常见疾病。

肺癌患者术后负担重，生活质量受到严重影响，对患者的结局指标测评尤为重要，近年来随着“以患者为中心”的照护理念不断深入，PRO逐渐成为结局评估的重要工具，并用于疾病进展的监测和临床治疗效果的评估。肺癌症状量表（Lung Cancer Symptom Scale, LCSS）经常被用来从身体和功能方面分析患者生活质量；欧洲癌症研究与治疗组织的生活质量核心量表（European Organization for Research and Treatment of Cancer Quality of Life Questionnaire, EORTC QLQ-C30）和肺癌特异模块（EORTC QLQ-Lung Cancer Module 13, EORTC QLQ-LC13）二者联用，即EORTC QLQ-Lung Cancer 43（EORTC QLQ-LC43），可全面评估肺癌患者生活质量。目前，EORTC QLQ-LC43被认为是最方便、最精准也是最常用的肺癌患者生活质量测量工具^[26]^；MD安德森症状清单肺癌模块（MD Anderson Symptom Inventory Lung Cancer Module, MDASI-LC）与核心量表联用可测量患者短期症状强度及对日常活动的干扰，较为方便简洁，经修订后具有较好的校标效度；肺癌患者术后症状量表聚焦肺癌围手术期患者及术后急性期症状，由于开发时间短，尚未广泛应用。

食管癌的首选治疗方法是手术，手术带来的创伤、术后并发症、不良反应等严重影响患者远期预后，PRO作为量化患者自身体验的高价值衡量标准，逐渐受到广泛关注，并为理论研究提供数据基础。癌症治疗功能评估--食管癌（Functional Assessment of Cancer Therapy-Esophageal, FACT-E）是用于评估食管癌患者整体生活质量的特异性PRO工具，但由于汉化时间较晚未能在国内广泛应用。

肺癌和食管癌等胸部常见肿瘤有较高的发病率，随着医疗水平的进步，患者生存期延长，治疗过程漫长，疾病不同时期的治疗手段、治疗地点等有了更多的选择，医患双方的沟通、患者的依从性等问题更需得到重视，SDM逐渐得到重视和应用。SDM可以让患者更清楚地了解不同治疗方法的具体情况，平衡医患双方信息差，让患者参与到治疗方式的选择中，提高患者的依从性，同时也减轻医生的责任负担，减少医患矛盾^[27]^。肺癌和食管癌的病情复杂，处于疾病不同时期的患者其需求和治疗方式不尽相同，SDM可以更精准地聚焦不同患者的关注点，帮助制定个性化诊疗方案，减少医患纠纷，更易取得患者的长期配合，有利于提高治疗效果和患者生活质量^[28]^。

近年来PRO和SDM的关注度不断增加，相关领域的研究快速增长，在国外尤其发达国家应用较多，我国则尚处于起步阶段。目前欧美等发达国家对PRO的研究较为成熟，形成了丰富的成熟量表，并广泛用于临床实践和诊疗^[29]^。我国对PRO的研究较少，使用的PRO工具多为从国外引进的量表进行翻译，对我国患者的适用度和信效度尚未达到十分令人满意的程度。尽管近年多位国内学者修订或设计更符合我国国情的量表，但仍缺少足够的信效度试验，尚未得到广泛应用。SDM同样在欧美地区发展较为成熟，我国则尚处于理论借鉴和应用摸索阶段。SDM在欧美国家和地区已有近半个世纪的研究历史，相关理论体系较为完善，开发了多种SDM评估工具、患者决策辅助工具。我国当前对SDM的研究还处于初步阶段，体现在诊疗决策等处，涉及的范围有限，相关研究局限在心血管、肿瘤等疾病，缺乏多科室、本土化的研究^[30]^。

患者报告结局测量信息系统（Patient-reported Outcomes Measure Information System, PROMIS）由美国牵头研制，是当前国际公认的最精确可信的PRO标准化测量工具系统^[31]^，在国外PROMIS被嵌入医院的信息系统，这也是大多数PROMIS电子化的应用方式，例如电子病历系统中嵌入PROMIS能更好地评估患者情况。除此之外，也有将PROMIS嵌入健康相关APP的应用方式。我国对PROMIS的应用还处于探索阶段，目前对其的应用多局限于儿童PROMIS。适用于中国患者人群的PROMIS还有待进行测量学检验和进一步研发，相关研究和数据较少，将PROMIS融入医院信息系统也尚需促进。

患者决策辅助工具（patient decision aid, PDA）是SDM实施的有效手段。PDA可以为患者提供各种治疗益处和危害信息，提高患者对疾病知识的认知，帮助患者更好地参与决策。在国外，PDA相关理论体系、政策法律已经相对成熟，PDA得到广泛应用，而我国目前PDA相关研究尚处于起步阶段，已开发的PDA种类较少，缺少本土化的高质量PDA，且未能真正应用在临床实践。

### 2.3 PRO与SDM的整合策略

将PRO和SDM的优点结合起来，可以构建一个更为全面和以患者为中心的治疗策略。在这种综合策略中PRO提供了一种量化和监测患者体验的方法，而SDM则确保了患者在治疗过程中的主动参与和决策权。要有效结合PRO和SDM，医疗团队可以在诊疗过程的早期就开始收集PRO数据，包括患者的症状、生活质量和治疗偏好等信息，这些数据随后可以在SDM的框架下与患者进行讨论，最大程度地确保患者的声音被听到并纳入到决策过程中。这种做法可以弥补SDM中可能存在的信息不对称问题，因为患者能够通过PRO直接地反馈自身的健康状况^[32]^。综合PRO和SDM的策略在提高治疗效果和患者满意度方面具有巨大潜力，通过PRO医生可以更精确地了解患者的状况，并及时调整治疗计划以满足患者的具体需求，而SDM则促进了医患之间的信任和沟通，使患者更加积极地参与到治疗中，从而提高依从性和预后。

然而，实施这一综合策略同样面临着挑战：（1）收集和分析PRO数据需要时间和资源，而且医疗团队需要接受相关培训以确保收集到的数据能够被有效使用；（2）SDM在实践中需要医生投入更多的时间和精力，与患者进行深入的沟通和讨论；（3）医疗系统中存在的固有流程和文化障碍也可能限制了PRO和SDM的广泛应用^[33]^。

为了解决这些挑战，可以通过开发和引入更高效的PRO数据收集和分析工具，积极采用互联网医院线上线下一体化的相关平台来减少医疗团队的负担^[34,35]^。同时，医疗机构可以提供SDM培训，以增强医生的沟通技巧。最重要的是，医疗政策制定者和管理者需要认识到综合PRO和SDM策略的价值，并在医疗体系中为此类实践创造更有利的环境。通过这些努力，综合PRO和SDM的策略有望在胸外科以及更广泛的医疗领域中，为患者提供更优质、更个性化的治疗体验。

## 3 总结和展望

在胸外科领域，PRO和SDM已被证明是两种极其重要的工具^[36]^。PRO允许医疗团队从患者的视角了解治疗效果，关注患者的生活质量和个人偏好，而SDM则强调了医患之间的对话，使患者能够在了解所有相关信息的基础上参与到决策过程中，从而选择与其价值观和生活目标最为一致的治疗方案。综合应用PRO和SDM不仅能够提升患者满意度和治疗依从性，还有助于优化治疗结果，减少不必要的医疗干预。

未来的研究方向应该集中在如何更有效地集成PRO和SDM到临床实践中，包括开发更为精细化的工具来收集和分析PRO数据^[37]^，以及设计更加高效的流程来促进医患之间的沟通。此外，研究应关注如何在不同的文化、经济和医疗体系背景下实施这些策略，以及如何针对不同患者群体定制化SDM过程。实践应用中的改进应包括提高医疗专业人员在PRO和SDM方面的培训，强化医疗系统对这两种方法的支持，以及探索利用数字化技术来简化数据收集和分析过程。同时，还需要进一步研究如何在医疗保险和支付模式中整合PRO和SDM，以确保医疗实践能够得到必要的资源支持。

总之，PRO和SDM在胸外科的应用代表了医疗服务以患者为中心的转变。通过不断优化两种策略的综合应用、相辅相成，有望在提高患者生活质量的同时，也提升整体的医疗效率。
